# Tumoral melanosis: A case series of patients with metastatic melanoma after systemic immunotherapy

**DOI:** 10.1016/j.jdcr.2024.01.011

**Published:** 2024-01-23

**Authors:** Sophia N. Wix, Meghan Heberton, Travis W. Vandergriff, Kim B. Yancey, Jennifer G. Gill

**Affiliations:** Department of Dermatology, University of Texas Southwestern Medical Center, Dallas, Texas

**Keywords:** dermal melanosis, immunotherapy, melanoma, melanophage, tumoral melanosis

## Introduction

Tumoral melanosis (TM) was first described in 1988 by Piérard and in 1994 by Barr as melanophage deposition within regressed melanoma tumors^1^ and, in subsequent decades, was reported infrequently in case reports.^2^, ^3^, ^4^ Regressed melanoma has been compared to TM and seen as a possible antecedent lesion to TM with distinguishable histopathologic features.^5^ Since 2015, the use of immunotherapy in melanoma patients has dramatically increased and led to the unprecedented survival of many advanced-stage patients. In parallel, TM has been increasingly reported in patients with metastatic melanoma (MM) after immunotherapy.^6^, ^7^, ^8^ TM can be challenging for dermatologists to manage since cutaneous TM lesions may closely resemble in-transit or subcutaneous metastases, and patients with MM often present to clinic with concerns of disease progression. While TM has been discussed in case reports^2^, ^3^, ^4^^,^^6^, ^7^, ^8^ and the histopathologic literature,^9^ its clinical features and natural history remain poorly described. Here we aim to better define cutaneous TM, outline the natural evolution of TM lesions, and offer guidance for management.

## Case series

This retrospective case series was performed at University of Texas Southwestern (UTSW) Medical Center (IRB STU-2022-0690). Patients were identified by the term “melanosis” via histopathologic data (*n* = 7) or clinical notes (*n* = 2) from a query of UTSW’s electronic medical record, regardless of age, race, or ethnicity, between January 2008 and October 2022. Patients with a history of melanoma of any stage and diagnosis of TM were included. Patients with nonmalignant diagnoses (eg, melanosis coli or vulvar melanosis) were excluded. Data extracted included: clinical descriptions and photographs of TM, pathology reports, imaging results, melanoma treatments, natural history of TM, and patient outcomes.

We identified 9 patients at UTSW with melanoma who developed cutaneous TM. All developed TM in 2017 or later and had a history of stage III or IV disease treated with systemic immunotherapy. The prevalence is estimated to be 0.83% (9/1083) of patients with malignant melanoma at UTSW since 2017 or 7.4% (9/122) of patients with melanoma who had an initial diagnosis of stage III or IV disease and initiated immunotherapy there. Of the 9 patients identified, the median age at first diagnosis of melanoma was 68 (range: 34-88) years with 5 females and 4 males (Table I). All received systemic immunotherapy prior to TM onset, including monotherapy or combinations of pembrolizumab, ipilimumab, and/or nivolumab. Patient 2 received talimogene laherparepvec after pembrolizumab. No other patients received talimogene laherparepvec. None of these patients underwent *BRAF*- or *MEK*-targeted therapies. Total immunotherapy duration varied with a median of 17.1 (range: 2.1-51.9) months (Table I). Other treatments included surgery, radiation, and epacadostat. One patient received palliative chemotherapy. The median time to TM diagnosis after the first dose of immunotherapy was 14.6 (range: 7.3-27.8) months, regardless of agent (Table I). Length of follow-up with dermatology after first TM detection was a median of 28.5 (range: 0.0-59.9) months (Table I).

**Table I:** Clinical features of 9 patients with metastatic melanoma that developed tumoral melanosis after immunotherapy

ID	Age at melanoma dx, decade/Sex	ICI received (duration, mo)	Location of primary melanoma(s)	Metastatic site(s)	Location of TM	No of TM lesions	Description of TM lesions	Time to TM dx after first ICI dose (mo)	Length of follow-up with derm since TM dx (mo)	Response to ICI∗	Survival/current clinical status
1	70s M	Ipi (18), pembro (34)	R upper arm	Cutaneous, LN	Regional; surrounding primary site and extending distally on UE	33	Blue-black to gray macules and papules	15.0	49.2 (ongoing)	PD (ipi); PD (pembro)	Alive on maintenance pembro
2	60s M	Pembro (8), T-VEC (9)	R upper arm	Cutaneous	Regional; surrounding primary site and extending distally on UE	53	Grouped, round black macules and thin papules	16.8	0	PR (pembro); CR (T-VEC)	Deceased due to unrelated causes after NED
3	60s F	Pembro-epacadostat (13), pembro (11), ipi (2)	Periurethral, L shoulder, L scalp	Adrenal, brain, cutaneous, liver, LN	Regional and distant; ipsilateral periauricular skin, R and L scalp skin	13	Blue-black and black round macules and dermal nodules	14.2	26.8	PD (pembro-epacadostat); PR (pembro); PD (ipi)	Deceased due to PD
4	30s M	Ipi-nivo (2), nivo (20)	L ear	Bone, brain, cutaneous, ocular	Distant; R upper arm, R flank, mid-back, R jaw, R eyelid	5	Blue-black dermal nodules, hypopigmented rim	27.8	28.5 (ongoing)	CR	Alive with NED
5	60s F	Ipi-nivo (2)	MUP	Brain, cutaneous, LN	NA/MUP; R shoulder, L elbow	2	Black dermal nodules	14.6	29.9 (ongoing)	CR	Alive with NED
6	80s F	Nivo (11)	R scalp	Cutaneous	Regional; surrounding primary site on R scalp	15	Grouped black macules	13.3	NA	CR	Alive with NED
7	70s F	Pembro (12)	L lower leg	Cutaneous, LN	Regional; ipsilateral thigh	1	Blue-black dermal nodule	11.9	NA	CR	Alive with NED
8	50s M	Pembro (11), ipi (2)	L upper back	Adrenal, bone, cutaneous, LN	Regional; surrounding primary site on L upper back	44	Scattered blue-black macules and nodules	7.3	59.9 (ongoing)	SD (pembro); CR (ipi)	Alive, declined further imaging after NED
9	70s F	Pembro (24)	L thigh	Adrenal, cutaneous, LN	Regional; scattered around primary on upper L thigh	8	Blue-black, deep green nodules	17.0	9.7 (ongoing)	PR	Alive on treatment holiday after disease control

*CR*, Complete response; *Derm*, dermatology; *Dx*, diagnosis; *F*, female; *ICI*, immune checkpoint inhibitor; *ID*, identifier; *Ipi*, ipilimumab; *L*, left; *LN*, lymph node; *M*, male; *Mo*, months; *MUP*, melanoma of unknown primary; *NA*, not available; *NED*, no evidence of disease; *Nivo*, nivolumab; *No*, number; *PD*, progressive disease; *Pembro*, pembrolizumab; *PR*, partial response; *R*, right; *SD*, stable disease; *TM*, tumoral melanosis; *T-VEC*, talimogene laherparepvec; *UE*, upper extremity.

∗ Following Response Evaluation Criteria in Solid Tumors Guidelines.^10^

**Fig 1:**
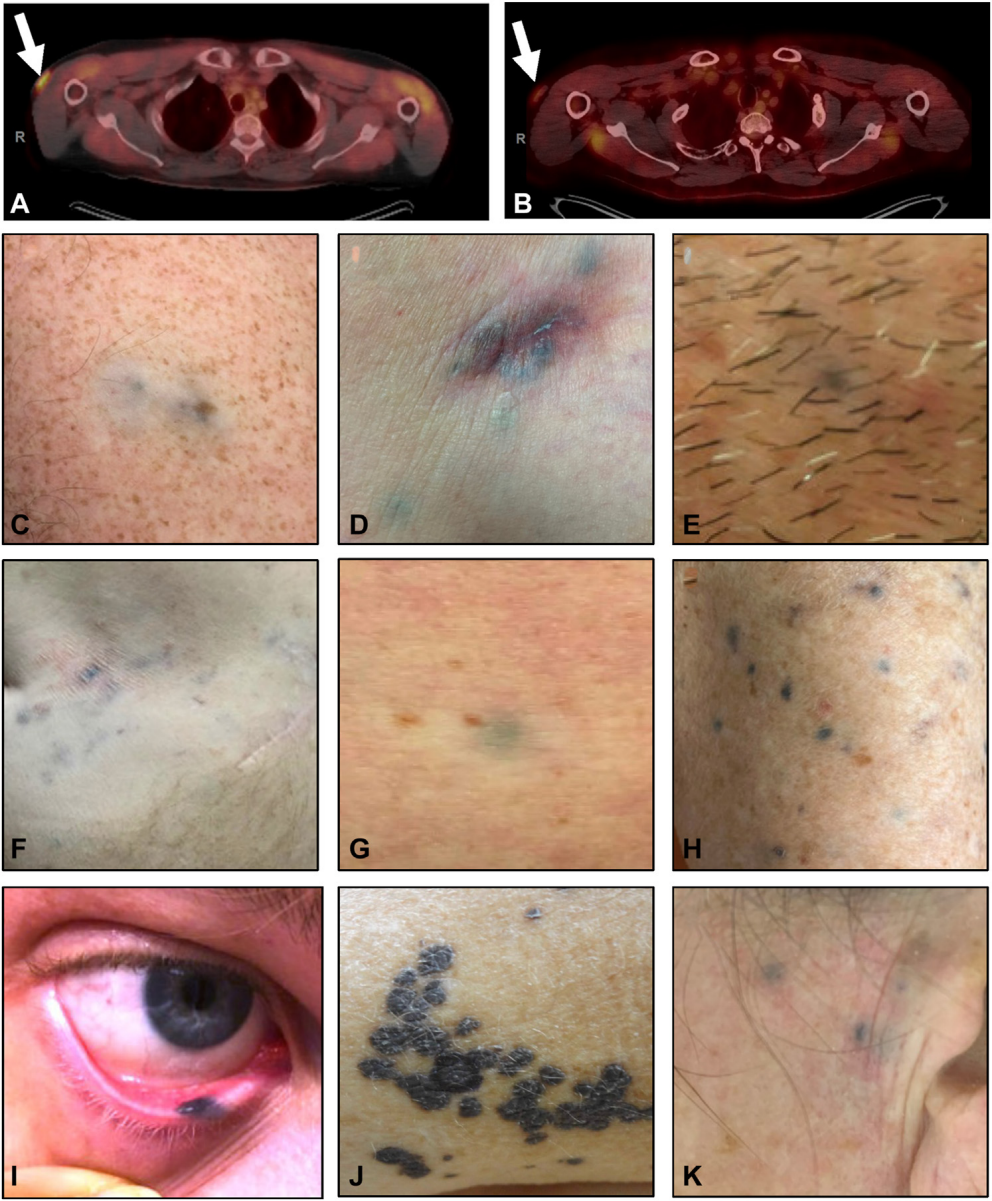
Varying clinical presentations of tumoral melanosis after immunotherapy in patients with metastatic melanoma and correlative findings on positron emission tomography-computed tomography (PET-CT). **A**-**C,** Sequential PET-CT images of a metastatic melanoma nodule (*white arrow*) that went from high fluorodeoxyglucose (FDG)-avidity (**A**) to normal FDG-avidity (**B**) 1.5 years after the start of immunotherapy, resulting in biopsy-proven tumoral melanosis pictured in (**C**). **D**-**K,** Varying clinical presentations of tumoral melanosis in patients with metastatic melanoma after systemic immunotherapy at our institution.

In 8 of 9 patients (89%), TM lesions arose in the context of previously detected cutaneous metastases (often biopsy-proven) or within lesions that were previously fluorodeoxyglucose (FDG)-avid on positron emission tomography-computed tomography (PET-CT) (Fig 1, *A*–*C*). TM presented as blue-gray, deep green, or black macules, papules, or nodules varying in depth and size (Fig 1, *C*–*K*). Some lesions also exhibited a depigmented rim, reminiscent of halo nevi (Fig 1, *C*). Importantly, sites of biopsy-proven TM demonstrated no significant FDG-avidity (Fig 1, *B*), despite prior high FDG-avidity when melanoma was active at that site (previously a subcutaneous metastasis) (Fig 1, *A*).

**Fig 2:**
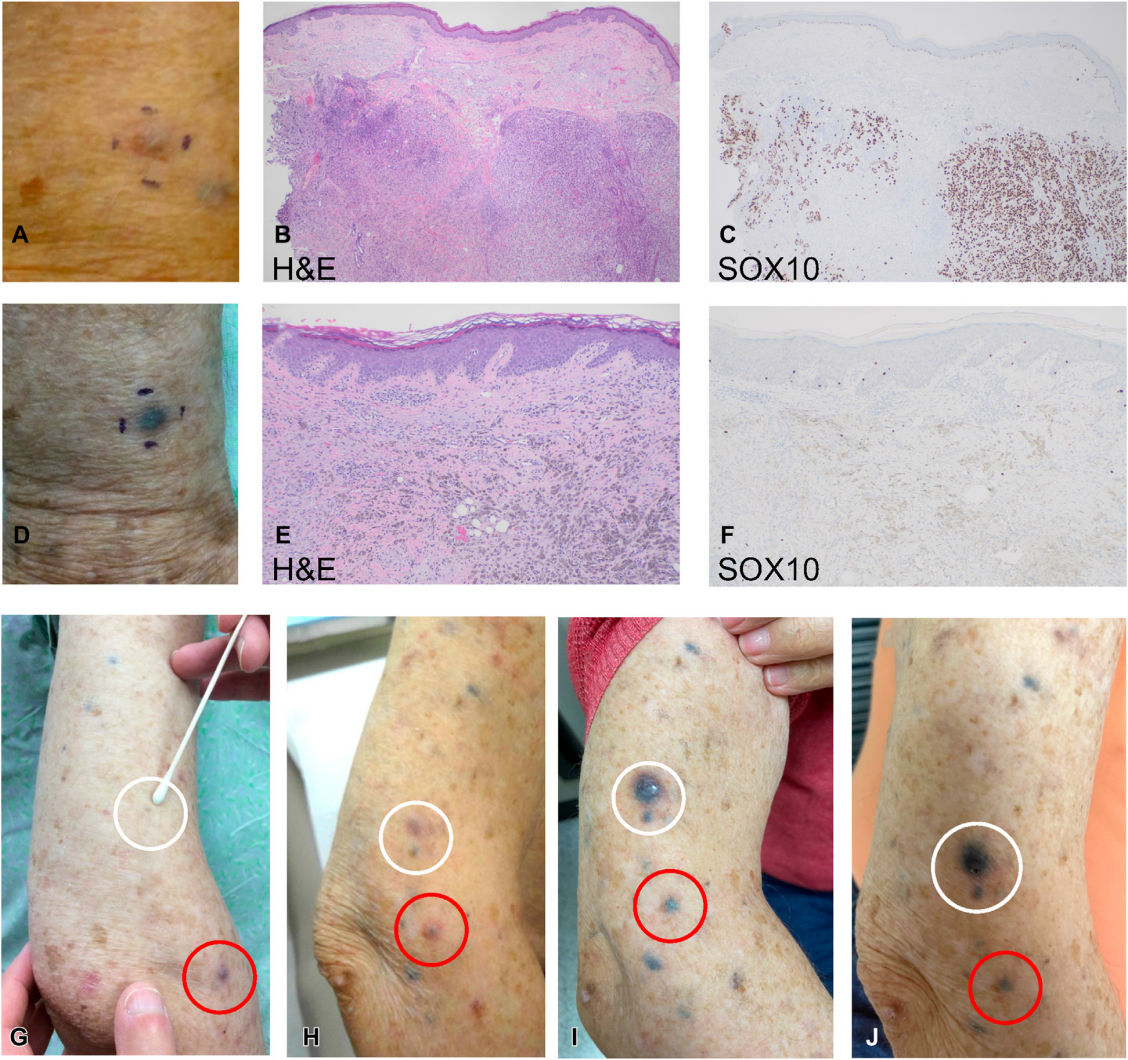
Histopathologic features and clinical evolution of tumoral melanosis (TM) in a patient with metastatic melanoma. **A,** Site of an active subcutaneous melanoma metastasis displaying early features of inflammation in patient 1. **B,** Hematoxylin and eosin (H&E) at 40× original magnification of the melanoma metastasis pictured in (**A**) with sparse nodular lymphocytic inflammation, sheets of atypical melanocytes, and melanophages in areas of fibrosis. **C,** Immunohistochemistry (IHC) at 40× original magnification demonstrating nuclear SOX10-positive cells consistent with active melanoma. **D,** Site of TM in patient 1. **E,** Histopathologic features of TM on H&E at 100× original magnification demonstrating dermal melanophages and fibrosis. **F,** IHC at 100× original magnification demonstrating a negative nuclear SOX-10 stain, indicating the lack of viable melanoma cells. **G**-**J,** Clinical evolution of 2 TM lesions (*circled in red and white*) on the aforementioned patient’s right upper arm after initiation of pembrolizumab and transition through TM stages of active melanoma (**G**), early evolving TM (**H**), late evolving TM (**I**), and stable TM (**J**). Images (**H**-**J**) were captured at 3-week intervals.

Of patients with known primaries, 7 of 8 (88%) developed at least 1 TM lesion in the same anatomic region as the primary (Table I). Patients developed varying numbers of TM lesions with a median of 13 (range: 1-53) lesions (Table I). Histologically, TM is characterized by dermal CD68-positive melanophages with largely negative nuclear SOX10 staining (Fig 2, *D*–*F*), in contrast to melanoma which demonstrates neoplastic cells with positive nuclear SOX10 (Fig 2, *A*–*C*).

All but Patient 7 developed systemic immune-related adverse events after immunotherapy, ranging from dermatitis and vitiligo to adrenal insufficiency and pneumonitis (data not shown). On follow-up, 8 of 9 (89%) patients ultimately achieved melanoma disease control after immunotherapy with a median follow-up of 3.7 (range: 1.4-5.9) years since immunotherapy initiation. Of these, 6 demonstrated complete response according to Response Evaluation Criteria in Solid Tumors Guidelines^10^ (Table I). One patient (11%) demonstrated fatal disease progression (Table I).

Of interest, patient 1 continues to have relapsing-remitting MM with ongoing TM development. The patient remains on pembrolizumab (duration of 34 months), and although he continues to develop new cutaneous metastases, these lesions subsequently evolve into TM within a few weeks (Fig 2, *G*–*J*). Lesions begin as small, palpable nodules, become inflamed and erythematous (Fig 2, *G*) with histologically detectable melanoma (Fig 2, *H* and *I*), and subsequently develop into darkly pigmented TM (Fig 2, *J*) containing only melanophages (Fig 2, *E* and *F*). He has been followed closely by dermatology with serial photography for 4.1 years and has not had melanoma recurrence within prior sites of TM or developed distant disease.

Notably, 4 of 9 (44%) patients, including patient 1, experienced melanoma recurrence or progression after the time of first TM detection, regardless of overall outcome. Patient 7 exhibited active biopsy-proven metastatic disease concurrently with biopsy-proven TM. No patients developed melanoma recurrence within existing TM lesions. TM lesions remained asymptomatic except in patient 4 who experienced local soreness and black exudative drainage 1 week after punch biopsy.

## Discussion

TM is an increasingly common cutaneous finding of patients with MM in the era of immunotherapy. Because TM resembles and follows cutaneous metastases, patients with MM often present to dermatology with concerns of disease progression.

We propose 4 stages of TM development: active melanoma, evolving TM, stable TM, and resolving TM. The first is “active” cutaneous melanoma metastases often within close anatomic proximity to the primary. Biopsy may reveal tumor-infiltrating lymphocytes, and metastases are hypermetabolic on PET-CT if large enough. The second stage is “evolving TM” which consists of metastases with associated erythema, increasing pigmentation, and decreasing volume. This lasts from a few weeks to months and correlates with decreased size and FDG-avidity (if applicable). “Stable TM” is characterized by dense dermal melanophages without evidence of residual melanoma on biopsy. Clinically, stable TM often appears as blue-gray, deep green, or black macules, papules, or nodules. In this case series, stable TM never increased in size, reacquired FDG-avidity, or displayed histopathologic features of active metastatic disease. In a subset of 4 patients with the longest dermatologic follow-up, some TM lesions were found to eventually fade or shrink over the course of years, known as “resolving TM” (data not shown).

Notably, although some patients experienced melanoma progression after their first TM diagnosis, no active melanoma recurred in sites of stable or resolving TM based on close physical exam monitoring, biopsies, and/or standard-of-care PET-CT imaging. We also observed active visceral or cutaneous metastases concurrently with biopsy-proven TM, demonstrating these states can coexist.

This case series has generated clinical curiosities that require further examination at the molecular level among larger cohorts. First, while some active cutaneous metastases were pigmented, some did not become pigmented until the “evolving,” inflammatory TM stage. Because specific cytokines can stimulate melanogenesis,^12^ we speculate this burst of melanogenesis prior to successful immune destruction may be a clue to the components of an effective antitumor response. It is worth noting our screen did not identify any patients who developed TM after *BRAF*-targeted therapy. While TM related to *BRAF*-targeted agents has been reported previously,^9^^,^^11^ we suspect this is less common due to tumor-intrinsic growth factor-mediated cell death, as opposed to immune-mediated destruction.

Although prior literature suggests biopsy is essential for TM management,^9^ we propose active monitoring with dermatology and serial photography as alternatives for patients with numerous lesions once an initial diagnosis is histologically confirmed. Further studies are needed to determine the role of mole-mapping technologies to track TM over time. For patients already undergoing serial PET-CT for treatment monitoring or restaging, PET-CT can also be used by dermatologists as a complementary tool alongside a thorough skin exam to distinguish active metastases from TM. It should be emphasized, however, that this tool should be reserved for tracking previously FDG-avid lesions, as not all active cutaneous metastases are large enough to appear hypermetabolic on PET-CT. Biopsy should be considered for any sites that are new, inflamed, nodular, FDG-avid, or growing as these all raise concern for active melanoma.

TM has been proposed to be a favorable prognosticator for melanoma after immunotherapy.^7^^,^^13^ Overall, 8 of 9 patients (89%) in our cohort achieved disease control, 6 of which achieved complete response. Notably, some patients progressed after TM development and ultimately required alternative immunotherapy agents to achieve disease control. This underscores the importance of close dermatologic monitoring of all advanced patients with melanoma. Larger, multicenter studies are needed to determine the clinical utility of TM as a reliable prognosticator.

This study is limited by its retrospective design. It is likely some cases of cutaneous TM from our institution were not captured in this cohort due to varied nomenclature among dermatopathologists and clinicians’ terminology in documentation. Also, we have only reported cases of cutaneous TM. Our study does not explore the characteristics of extracutaneous TM, such as TM found within visceral organs or lymph nodes, as previously reported in other case studies.^14^^,^^15^ Because the first documented case of TM at our center was in 2017, longer-term patient outcomes are not yet available. As the incidence of TM increases in frequency with the widespread use of immunotherapy for MM, larger cohorts and multi-institutional studies will be needed to better understand the natural history of TM, its evolution in other treatment settings, and prognostic potential.

## Conflicts of interest

None disclosed.
